# Effect of Spinal Anesthesia versus General Anesthesia on Blood Glucose Concentration in Patients Undergoing Elective Cesarean Section Surgery: A Prospective Comparative Study

**DOI:** 10.1155/2019/7585043

**Published:** 2019-10-01

**Authors:** Khaled El-Radaideh, Ala”a Alhowary, Mohammad Alsawalmeh, Ahmed Abokmael, Haitham Odat, Amer Sindiani

**Affiliations:** ^1^Department of Anesthesiology and Intensive Care, Faculty of Medicine, Jordan University of Science and Technology, P.O. Box 953, Irbid 21110, Jordan; ^2^Department of Special Surgery, Faculty of Medicine, Jordan University of Science and Technology, P.O. Box 953, Irbid 21110, Jordan; ^3^Department of Obstetrics & Gynecology, Faculty of Medicine, Jordan University of Science and Technology, P.O. Box 953, Irbid 21110, Jordan

## Abstract

**Background:**

This prospective study compared the blood glucose concentration with spinal anesthesia or general anesthesia in patients undergoing elective cesarean section surgery.

**Methods:**

In total, 58 pregnant women who underwent elective cesarean section surgery were included in this prospective comparative study. Group S (*n* = 35) included patients who chose spinal anesthesia, and group G (*n* = 23) included patients who chose general anesthesia. The patients were allocated to the groups upon patients' preference. For the group G, the blood glucose concentration (BGC) was obtained 5 minutes before induction, T1, and 5 minutes after induction T2. For the group S, the BGC was obtained immediately before the injection of the local anesthetic agent T1 and 5 minutes after the complete block T2. For both groups, BGC was measured 5 minutes before the end of surgery T3 and 30 minutes after the end of surgery T4. For BGC measurements, we used a blood glucose monitoring system with a lancet device to prick the finger.

**Results:**

There was no statistically significant difference in the mean blood glucose concentration between the groups S and G in T1 (78.3 ± 18.2 vs. 74.3 ± 14.7, *p* > 0.05) and T2 (79.2 ± 18.3 vs. 84.9 ± 23.7, *p* > 0.05). The mean BGC was statistically significantly higher in group G in comparison to group S in the times 5 minutes before (80.2 ± 18.1 vs. 108.4 ± 16.7, *p* < 0.05) and 30 minutes after the end of surgery (80.9 ± 17.7 vs. 121.1 ± 17.4, *p* < 0.05).

**Conclusion:**

There is a much lower increase in blood glucose concentration under spinal anesthesia than under general anesthesia. It is reasonable to suggest that the blood sugar concentration must be intraoperatively monitored in patients undergoing general anesthesia.

## 1. Introduction

Spinal anesthesia has become the preferred anesthetic technique when providing anesthesia for patients undergoing elective cesarean section as the risk of maternal and fetal complications associated with spinal anesthesia is less than with general anesthesia [1–5].

Every surgical procedure is associated with a stress response which comprises a number of endocrine, metabolic, and immunological changes triggered by neuronal activation of the hypothalamic-pituitary-adrenal axis [6, 7]. The overall metabolic effect of the stress response to surgery includes an increase in secretion of catabolic hormones, such as cortisol and catecholamine, and a decrease in secretion of anabolic hormones, such as insulin and testosterone. The increase in levels of catabolic hormones in plasma stimulates glucose production, and there is a relative lack of insulin together with impaired tissue insulin sensitivity and glucose utilization, which is called insulin resistance. Consequently, blood glucose concentrations will increase, even in the absence of preexisting diabetes [6–9].

The hyperglycemic response to surgical stresses in the perioperative period may harm patients since it is an independent risk factor associated with adverse outcomes such as impaired wound healing and an increased risk of wound infection [7, 9, 10]. The risk related to hyperglycemia is seen in patients both with and without a history of diabetes [11]. Notably, even short-term hyperglycemia compromises immune function and increases the risk of infection [9, 12, 13].

In surgical patients, the stress response is activated by afferent neural activity from the site of trauma. These afferent neurons travel along sensory nerve roots through the dorsal root of the spinal cord up the spinal cord to the medulla to activate the hypothalamus. Neuraxial anesthesia such as epidural or spinal anesthesia blocks afferent neural impulses; consequently, the stress response to surgery including hyperglycemia is inhibited [6, 7, 14, 15].

In the present study, we tested the hypothesis that spinal anesthesia would result in a less-pronounced stress-induced hyperglycemia than general anesthesia during cesarean sections in nondiabetic patients.

## 2. Materials and Methods

After obtaining formal approval from the institutional ethics committee (approval no. 3492017), a prospective comparative study included 58 pregnant women scheduled for the elective cesarean section at King Abdullah University Hospital. Written informed consents for participation in the study were obtained from all patients.

The criteria for inclusion in this study were female patients who were scheduled for elective cesarean section, American Society of Anesthesiologists (ASA) physical status of I–II, above 18 years of age, and fasting time preoperatively between 8 and 12 hours. Patients with diabetes mellitus type 1, diabetes mellitus type 2, gestational diabetes, chronic advanced renal disease, heart failure, ischemic heart disease, eclampsia, preeclampsia, and psychiatric disorders were excluded. All patients with failed spinal anesthesia and those who had converted to general anesthesia from spinal anesthesia were excluded.

On arrival at the operating theater, two intravenous access sites were prepared. For all participants in the study, standard monitoring of blood pressure, three-lead electrocardiogram, and pulse oximetry oxygen saturation were conducted and continuously monitored during the intraoperative period in the operating theater and during the postoperative period in the postanesthesia care unit.

The patients were electively allocated to two groups (S and G). Group S included patients who chose spinal anesthesia, and group G included patients who chose general anesthesia.

For group S, spinal anesthesia was administered under aseptic conditions, at the level of L3-L4 or L4-L5 of the spinal column. Spinal anesthesia was performed with 2.3 ml of 0.5% heavy bupivacaine and 0.4 ml of 0.005% fentanyl using 25- or 27-gauge spinal needles; 100% O_2_ was administered through a simple face mask with a flow of 4 liters per minute.

For group G, after breathing oxygen for 3 minutes via a face mask, anesthesia was induced with 2–2.5 mg/kg propofol and 0.6 mg/kg rocuronium to facilitate tracheal intubation and with rapid sequence intubation using a regular 6.5 mm ID endotracheal tube. After delivery of the baby and cutting the umbilical cord, 3 μg/kg fentanyl was given. Before delivery of the baby, anesthesia was maintained with 0.7% isoflurane in 50% oxygen and 50% nitrous oxide, and after delivery and cutting the umbilical cord, anesthesia was maintained with a propofol infusion at a rate of 150 *μ*g/kg/min and the inhaled anesthetic agents were discontinued. ETCO_2_ was maintained between 30 mmHg and 40 mmHg throughout the surgery. At the end of surgery, anesthetic maintenance was discontinued, and reversal of the neuromuscular blockade consisting of 2.5 mg of neostigmine and 1 mg of atropine was given intravenously (IV). The extubation of the trachea was performed when the patient was breathing spontaneously with a good tidal volume, fully awake, and could sustain head elevation for more than 5 seconds.

Upon arrival at the operating theater, both groups received 750 mg of cefuroxime IV, 8 mg of dexamethasone IV, 50 mg of ranitidine IV, and 10 mg of metoclopramide IV before starting anesthesia. After delivery of the baby, both groups received 10 IU oxytocin IV bolus and 20 IU oxytocin infusion over 1 hour. Both groups were given 2000–3000 ml crystalloids IV; half of the amount was 0.9% normal saline, and the other half was Ringer's lactate solution.

For the group G, the blood glucose concentration (BGC) was obtained 5 minutes before induction (T1) and 5 minutes after induction (T2). For the group S, the BGC was obtained immediately before the injection of the local anesthetic agent (T1) and 5 minutes after the complete block (T2). For both groups, the blood glucose concentration was measured 5 minutes before the end of surgery, T3, and 30 minutes after the end of surgery in the postanesthesia care unit, T4, using a blood glucose monitoring kit with a lancet device (Joycoo BG-102; Joycoo, Amman, Jordan). After disinfecting with alcohol, swap the tips of the fingers of the nondominant hand pricked with a lancet tip to measure the blood glucose concentration.

### 2.1. Statistical Analysis

A sample size of 20 patients per group was required to achieve a power of 0.80 and alpha of 0.05 based on a hypothetical 25% increase in glucose concentration either at the end of surgery or after surgery. Mean age, weight, and fasting time were compared in group S and group G using the *t*-test. To test for statistically significant differences between the four blood glucose readings and their interaction with the type of anesthesia, repeated measures of analysis of variance were conducted and the results of such analyses are reported in Tables 1 and 2. Statistical analyses were performed using SPSS for Windows version 18.0 (SPSS Inc., Chicago, IL, USA). All values were expressed as mean ± SD unless otherwise specified, and *p* values <0.05 were considered to be statistically significant.

## 3. Results

The two groups were statistically equivalent with regard to age, weight, and fasting duration, as indicated in Table 3, which shows the means, standard deviations, and *t*-test statistic for the difference between the mean values.  Table 4 shows the means and standard deviations for the four glucose-check readings for the two groups. The mean values for the general anesthesia group increased more rapidly than those in the spinal anesthesia group.

According to Tables 1 and 2, there was a statistically significant difference (at *α* = 0.01) in glucose-check readings with regard to time of readings and its interaction with type of anesthesia (general anesthesia and spinal anesthesia).

**Figure 1:**
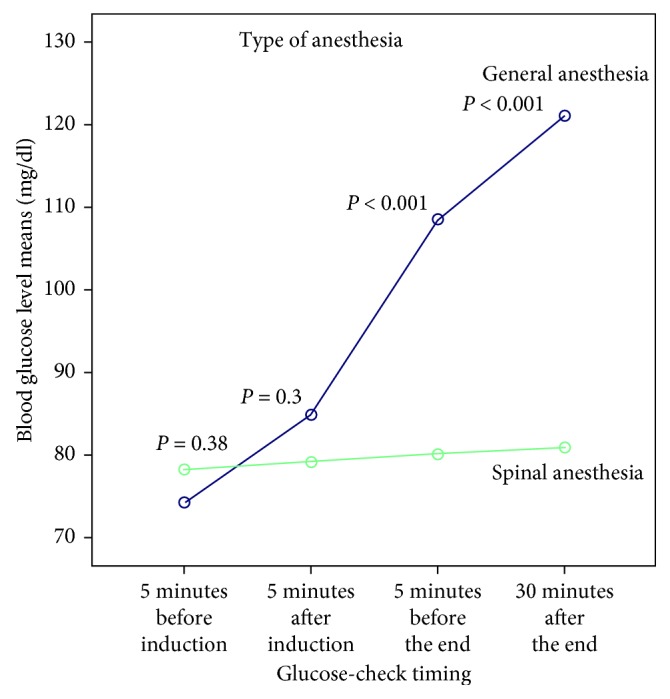
Relationship between glucose-check timing, type of anesthesia, and mean blood glucose concentrations for patients in the study.

The results in Table 4 were plotted as a graph in Figure 1. This shows the difference between the glucose-check readings according to glucose-check timing in both spinal anesthesia and general anesthesia and shows the difference in the effect of type of anesthesia (general anesthesia and spinal anesthesia) on blood glucose concentration. According to Figure 1, there is a significant proportional increase in mean blood glucose concentrations with glucose-check timing (5 minutes before induction, 5 minutes after induction, 5 minutes before the end of surgery, and 30 minutes after the end of surgery), and this increase is significantly much greater in general anesthesia than it is in spinal anesthesia.

## 4. Discussion

This study compared the effects of spinal and general anesthesia on changes in blood glucose concentrations during cesarean section in nondiabetic patients. Although mean blood glucose concentrations showed a significant proportional increase during surgery in both groups, this effect was much more significant with general anesthesia than with spinal anesthesia. These results indicate that spinal anesthesia is more effective than general anesthesia in attenuating the hyperglycemic response to surgery during cesarean section.

There has been a great deal of interest in the potential beneficial effects of preservation of glucose homeostasis and early avoidance of stress-induced hyperglycemia in surgical patients by modification of the stress response. Acute hyperglycemia, a typical feature of the metabolic response to surgery, has been demonstrated to significantly compromise immune function and contributes to poor clinical outcome [9, 12, 13, 16]. The degree of this response was shown to be proportional to the severity and length of the surgical injury [17], and the magnitude of insulin resistance increased during surgery according to the degree of surgical injury [11]. Turinaet al. [12] showed that short-term hyperglycemia is associated with increased risk of infection and mortality in critically ill patients related to a significant decrease in monocyte HLA-DR expression due to hyperglycemia and hyperinsulinemia.

Treating hyperglycemia results in an increased risk of hypoglycemia and the risks associated with hypoglycemia, and thus avoidance of stress-induced hyperglycemia is preferable for treating dysglycemia [15]. It has long been recognized that the type of anesthetic technique has an influence on hyperglycemic response to surgery [18]. During surgery, stress-induced hyperglycemia is more pronounced with inhalation anesthesia. In animals, earlier studies revealed that inhalational anesthetics such as enflurane and halothane impaired glucose tolerance in dogs and that was related to inhibition of insulin secretion and decreased tissue insulin sensitivity [9]. Other studies on isoflurane inhalational anesthetic demonstrated an increase in the plasma glucose concentration during anesthesia even without surgical stress related to impairment of glucose tolerance and stimulation of whole body glucose production [9, 13, 19]. Furthermore, the hyperglycemic stress response in patients undergoing major abdominal surgery under isoflurane general anesthesia could be related to an increase in endogenous glucose production accompanied by a decrease in glucose utilization [9, 20] Tanaka et al. [21]showed that there was glucose intolerance and impairment of insulin secretion and glucose utilization during sevoflurane and isoflurane anesthesia in a dose-independent manner. According to the results of a study by Cok et al. [22], although isoflurane and propofol, both combined with remifentanil, provided a clinically comparable insulin and cortisol response to surgery in craniotomy operations, propofol attenuated the increase in plasma blood glucose. This suggested that propofol may be preferred over isoflurane when tight control of blood glucose is a goal.

Regarding neuraxial anesthesia such as epidural or spinal block with local anesthetics, this blocks both afferent input from the operative site to the central nervous system and the hypothalamic-pituitary axis and efferent autonomic neuronal pathways to the liver and adrenal medulla. Consequently, the adrenocortical and glycemic responses to surgery are greatly inhibited [7, 16]. A study by Kehlet [23] showed that epidural blockade attenuated the hyperglycemic response during surgery, most likely mediated through its inhibitory action on the hypothalamic-pituitary-adrenal axis. Some studies looking at glucose tolerance tests during pelvic procedures showed that epidural block improved tissue glucose uptake [24, 25]. In contrast, other studies revealed that epidural block attenuated the hyperglycemic response during surgery by inhibiting hepatic glucose release rather than improving tissue glucose utilization [26, 27]. In another study, Lattermannet al. [16] concluded that epidural blockade attenuated the hyperglycemic response to abdominal surgery through modification of glucose production without affecting glucose utilization. However, it is still unclear whether the inhibitory effect of the epidural block on the hyperglycemic response during surgery was a consequence of the improvement in tissue glucose uptake, a decrease in glucose production, or a combination of both. In any case, it has been well recognized that epidural blockade with a local anesthetic inhibits or even prevents the endocrine and metabolic responses to surgery including hyperglycemia [16]. An earlier study by Enquist et al. [28] showed that epidural blockade, established before the start of surgery, prevented the increase in plasma glucose and cortisol levels in response to surgery in patients undergoing hysterectomy. A more recent study by Hadimioglu et al. [6] demonstrated that combined general and epidural anesthesia, when compared with general anesthesia alone, reduced inflammatory activation and insulin resistance responses to the stress of the renal transplantation procedure and that inhibition of stress responses had a beneficial effect on length of stay in hospital postoperatively. To summarize those previously mentioned studies, epidural anesthesia attenuates the hyperglycemic response during surgery. Our study looked at spinal anesthesia, a different neuraxial technique, and confirmed the results of those earlier studies as spinal anesthesia resulted in effects comparable to epidural anesthesia with attenuation of the hyperglycemic response to surgery.

As we mentioned previously, spinal anesthesia is the most common technique used to provide anesthesia for patients undergoing elective cesarean section due to the lower risk of maternal and fetal complications associated with spinal anesthesia than general anesthesia. The results of our study add more weight to the use of spinal anesthesia in the obstetric population since spinal anesthesia facilitates glycemic control in the perioperative period. This might be beneficial in reducing the incidence of previously mentioned complications associated with hyperglycemia and other maternal and fetal complications. Therefore, these added benefits of spinal anesthesia over general anesthesia should be conveyed to patients during patient counseling about cesarean sections.

One study limitation is that our study design did not allow measurement of the level of stress hormones. Furthermore, we only measured blood glucose concentrations up to 30 minutes after the end of surgery; therefore, the long-term trends are not known. However, our study was not designed to address the long-term effects of spinal anesthesia on hyperglycemic response to surgery or to study the effects of spinal anesthesia on other parameters of stress response to surgery. Another limitation of our study is that it only included nondiabetic pregnant women who were candidates for elective cesarean section surgery. The results may be different in diabetic patients, in male patients, or in patients undergoing other types of surgery, and so further studies are required to examine the effects of spinal anesthesia on blood glucose concentrations among these groups.

In conclusion, according to this study, there was a significant proportional increase in mean blood glucose concentrations from glucose-check timing with both general anesthesia and spinal anesthesia. The effect of general anesthesia on blood glucose concentrations was significantly greater than the effect of spinal anesthesia, which indicates that the hormonal stress response is much greater in general anesthesia than in spinal anesthesia.

## Figures and Tables

**Table 1:** Multivariate tests^a^ on glucose-check data and type of anesthesia for patients included in the study.

Effect		Hypothesis				
		Value	*F*	df	Error df	Significance
Glucose-check	Wilks' lambda	0.168	88.838^b^	3.000	54.000	0.000
Glucose level *∗* type of anesthesia	Wilks' lambda	0.204	70.440^b^	3.000	54.000	0.000

^a^Design: intercept + type of anesthesia. Within-subjects design: glucose-check. ^b^Exact statistic.

**Table 2:** Tests of within-subjects effects for patients included in the study.

Measure: readings						
Source		Type III sum of squares	df	Mean square	*F*	Significance
Glucose-check	Sphericity assumed	21192.823	3	7064.274	131.448	0.000
Glucose level *∗* type of anesthesia	Sphericity assumed	17143.099	3	5714.366	106.329	0.000
Error (glucose-check)	Sphericity assumed	9028.673	168	53.742		

**Table 3:** Age, weight, and fasting time of patients included in the study (*n* = 58).

	Group G, mean ± SD (*n* = 23)	Group S, mean ± SD (*n* = 35)	*t*	*P* value
Age (years)	28.2 ± 4.2	28.9 ± 5.6	–0.470	0.640
Weight (kg)	69.4 ± 8.3	70.4 ± 12.5	–0.330	0.742
Fasting time (hours)	9.2 ± 1.4	9.7 ± 1.2	–1.584	0.119

Data are given as mean ± SD and the significance of the difference in age, weight, and fasting time between group G (general anesthesia) and group S (spinal anesthesia).

**Table 4:** Descriptive statistics for the mean blood glucose concentrations for spinal anesthesia and general anesthesia at different measurement times.

	Type of anesthesia	Mean	Std. deviation	*N*
5 min before induction	General anesthesia	74.3	14.7	23
	Spinal anesthesia	78.3	18.2	35
	Total	76.7	16.9	58

5 min after induction	General anesthesia	84.9	23.7	23
	Spinal anesthesia	79.2	18.3	35
	Total	81.4	20.6	58

5 min before the end of surgery	General anesthesia	108.4	16.7	23
	Spinal anesthesia	80.2	18.1	35
	Total	91.4	22.3	58

30 min after the end of surgery	General anesthesia	121.1	17.4	23
	Spinal anesthesia	80.9	17.7	35
	Total	96.8	26.4	58

## Data Availability

The data used to support the findings of this study have been deposited in the Figshare repository (DOI: https://doi.org/10.6084/m9.figshare.845654?noredirect).
